# 4-Chloro-*N*′-(4-meth­oxy­benzyl­idene)benzohydrazide methanol monosolvate

**DOI:** 10.1107/S1600536810038857

**Published:** 2010-10-09

**Authors:** Hai-Tao Huang, Hong-Yuan Wu

**Affiliations:** a Pharmacy School, Qiqihar Medical University, Qiqihar 161006, People’s Republic of China; b College of Chemistry and Chemical Engineering, Qiqihar University, Qiqihar 161006, People’s Republic of China

## Abstract

The title compound, C_15_H_13_ClN_2_O_2_·CH_4_O, consists of a 4-chloro-*N*′-(4-meth­oxy­benzyl­idene)benzohydrazide (CMB) mol­ecule and a methanol mol­ecule of crystallization. It was obtained by the condensation of 4-meth­oxy­benzaldehyde with 4-chloro­benzohydrazide. In the CMB mol­ecule, the dihedral angle between the two benzene rings is 50.1 (3)°. The methanol mol­ecule is linked to the CMB mol­ecule through O—H⋯O and O—H⋯N hydrogen bonds. In the crystal, CMB mol­ecules are linked through inter­molecular N—H⋯O hydrogen bonds, involving the methanol mol­ecule, forming chains propagating along [010].

## Related literature

For background to compounds obtained by the condensation of aldehydes with benzohydrazides, see: Qiu & Zhao (2008 ▶); Yathirajan *et al.* (2007 ▶); Salhin *et al.* (2007 ▶). For their biological properties, see: Bedia *et al.* (2006 ▶); Terzioglu & Gürsoy (2003 ▶); Küçükgüzel *et al.* (2003 ▶); Charkoudian *et al.* (2007 ▶). For similar compounds reported by our group, see: Huang (2009 ▶); Wu (2009 ▶). For other similar structures, see: Fun *et al.* (2008 ▶); Liu & Li (2004 ▶); Lei *et al.* (2008 ▶). For bond-length data, see: Allen *et al.* (1987 ▶).
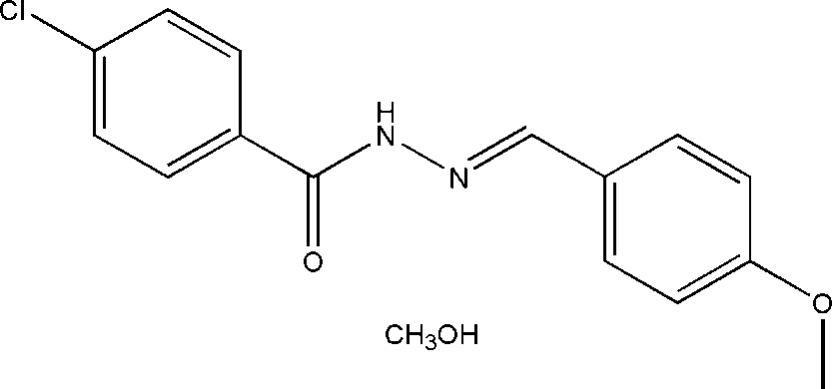


## Experimental

### 

#### Crystal data

C_15_H_13_ClN_2_O_2_·CH_4_O
*M* *_r_* = 320.77
Monoclinic, 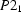

*a* = 10.914 (3) Å
*b* = 6.459 (2) Å
*c* = 11.358 (2) Å
β = 93.000 (3)°
*V* = 799.6 (4) Å^3^
*Z* = 2
Mo *K*α radiation
μ = 0.25 mm^−1^
*T* = 298 K
0.17 × 0.13 × 0.12 mm

#### Data collection

Bruker APEXII CCD area-detector diffractometer
Absorption correction: multi-scan (*SADABS*; Sheldrick, 1996 ▶) *T*_min_ = 0.958, *T*_max_ = 0.970
6424 measured reflections
1865 independent reflections
1030 reflections with *I* > 2σ(*I*)
*R*_int_ = 0.074

#### Refinement

*R*[*F* ^2^ > 2σ(*F* ^2^)] = 0.060
*wR*(*F* ^2^) = 0.123
*S* = 1.00
1865 reflections
201 parameters
1 restraint
H-atom parameters constrained
Δρ_max_ = 0.24 e Å^−3^
Δρ_min_ = −0.19 e Å^−3^

### 

Data collection: *APEX2* (Bruker, 2007 ▶); cell refinement: *SAINT* (Bruker, 2007 ▶); data reduction: *SAINT*; program(s) used to solve structure: *SHELXS97* (Sheldrick, 2008 ▶); program(s) used to refine structure: *SHELXL97* (Sheldrick, 2008 ▶); molecular graphics: *SHELXTL* (Sheldrick, 2008 ▶); software used to prepare material for publication: *SHELXTL*.

## Supplementary Material

Crystal structure: contains datablocks global, I. DOI: 10.1107/S1600536810038857/su2215sup1.cif

Structure factors: contains datablocks I. DOI: 10.1107/S1600536810038857/su2215Isup2.hkl

Additional supplementary materials:  crystallographic information; 3D view; checkCIF report

## Figures and Tables

**Table 1:** Hydrogen-bond geometry (Å, °)

*D*—H⋯*A*	*D*—H	H⋯*A*	*D*⋯*A*	*D*—H⋯*A*
O3—H3⋯N2	0.82	2.47	3.184 (6)	146
O3—H3⋯O1	0.82	2.12	2.820 (6)	143
N1—H1⋯O3^i^	0.86	2.08	2.880 (6)	154

Symmetry code: (i) 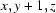.

**Fig. 1.**
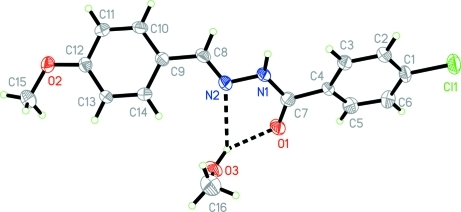
The molecular structure of the title compound, showing the atom-numbering scheme and displacement ellipsoids drawn at the 30% probability level. Hydrogen bonds are shown as dashed lines.

**Fig. 2.**
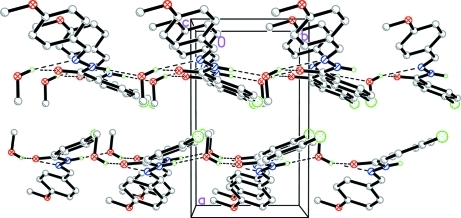
The crystal packing of the title compound viewed along the c axis. Hydrogen bonds are shown as dashed lines - see Table 1 for details (H-atoms not involved in hydrogen bonding have been omitted for clarity).

## supplementary crystallographic information

### Comment

In the last few years considerable attention has been focused on compounds derived from the condensation of aldehydes with benzohydrazides, especially for their crystal structures (Lei *et al.*, 2008; Qiu & Zhao, 2008; Yathirajan *et al.*, 2007; Salhin *et al.*, 2007; Fun *et al.*, 2008; Liu & Li, 2004) or for their biological properties (Bedia *et al.*, 2006; Terzioglu & Gürsoy, 2003; Küçükgüzel *et al.*, 2003; Charkoudian *et al.*, 2007). Continueing our research on the synthesis and crystal structures of such compounds (Huang, 2009; Wu, 2009), herein we report on the crystal structure of the title compound, obtained by the condensation of 4-methoxybenzaldehyde with 4-chlorobenzohydrazide.

The title compound consists of a 4-chloro-*N*'-(4-methoxybenzylidene)benzohydrazide (CMB) molecule and a methanol solvent molecule (Fig. 1). The methanol molecule is linked to the CMB molecule through intermolecular O—H···O and O—H···N hydrogen bonds (Table 1). In the CMB molecule the dihedral angle between the two benzene rings is 50.1 (3)°. The bond distances (Allen *et al.*, 1987) and bond angles are normal and similar to those reported for the above mentioned compounds.

In the crystal molecules are linked, *via* the methanol molecule, through intermolecular N—H···O hydrogen bonds (Table 1), so forming chains propagating along the *b* axis (Fig. 2).

### Experimental

The title compound was prepared by the condensation of 4-methoxybenzaldehyde (0.1 mol) and 4-chlorobenzohydrazide (0.1 mol) in ethanol (20 ml). The excess ethanol was removed by distillation. The colou; rless solid obtained was filtered and washed with ethanol. Single crystals, suitable for X-ray diffraction, were obtained on slow evaporation of a solution of the title compound in methanol.

### Refinement

As there is no asymmetric center in the title molecule in the final cycles of least-squares refinement 1371 Friedel pairs were merged and Δf" set to zero, rather than refining the structure as a inversion twin. The H-atoms were positioned geometrically and treated as riding atoms: O—H = 0.82 Å, N—H = 0.86 Å, C—H = 0.93 and 0.96 Å, for CH and CH_3_ H-atoms, respectively, with *U*_iso_(H) = k × *U*_eq_(O–, N–, C-parent atom), where k = 1.5 for OH and CH_3_ H-atoms and k = 1.2 for all other H-atoms.

### Crystal data

**Table:**

C_15_H_13_ClN_2_O_2_·CH_4_O	*F*(000) = 336
*M**_r_* = 320.77	*D*_x_ = 1.332 Mg m^−^^3^
Monoclinic, *P*2_1_	Mo *K*α radiation, λ = 0.71073 Å
Hall symbol: P 2yb	Cell parameters from 640 reflections
*a* = 10.914 (3) Å	θ = 2.5–24.3°
*b* = 6.459 (2) Å	µ = 0.25 mm^−^^1^
*c* = 11.358 (2) Å	*T* = 298 K
β = 93.000 (3)°	Block, colorless
*V* = 799.6 (4) Å^3^	0.17 × 0.13 × 0.12 mm
*Z* = 2	

### Data collection

**Table:**

Bruker APEXII CCD area-detector diffractometer	1865 independent reflections
Radiation source: fine-focus sealed tube	1030 reflections with *I* > 2σ(*I*)
graphite	*R*_int_ = 0.074
ω scans	θ_max_ = 27.0°, θ_min_ = 1.8°
Absorption correction: multi-scan (*SADABS*; Sheldrick, 1996)	*h* = −13→13
*T*_min_ = 0.958, *T*_max_ = 0.970	*k* = −7→8
6424 measured reflections	*l* = −14→14

### Refinement

**Table:**

Refinement on *F*^2^	Primary atom site location: structure-invariant direct methods
Least-squares matrix: full	Secondary atom site location: difference Fourier map
*R*[*F*^2^ > 2σ(*F*^2^)] = 0.060	Hydrogen site location: inferred from neighbouring sites
*wR*(*F*^2^) = 0.123	H-atom parameters constrained
*S* = 1.00	*w* = 1/[σ^2^(*F*_o_^2^) + (0.048*P*)^2^] where *P* = (*F*_o_^2^ + 2*F*_c_^2^)/3
1865 reflections	(Δ/σ)_max_ < 0.001
201 parameters	Δρ_max_ = 0.24 e Å^−^^3^
1 restraint	Δρ_min_ = −0.19 e Å^−^^3^

### Special details

**Table:**

Geometry. All e.s.d.'s (except the e.s.d. in the dihedral angle between two l.s. planes) are estimated using the full covariance matrix. The cell e.s.d.'s are taken into account individually in the estimation of e.s.d.'s in distances, angles and torsion angles; correlations between e.s.d.'s in cell parameters are only used when they are defined by crystal symmetry. An approximate (isotropic) treatment of cell e.s.d.'s is used for estimating e.s.d.'s involving l.s. planes.
Refinement. Refinement of *F*^2^ against ALL reflections. The weighted *R*-factor *wR* and goodness of fit *S* are based on *F*^2^, conventional *R*-factors *R* are based on *F*, with *F* set to zero for negative *F*^2^. The threshold expression of *F*^2^ > σ(*F*^2^) is used only for calculating *R*-factors(gt) *etc*. and is not relevant to the choice of reflections for refinement. *R*-factors based on *F*^2^ are statistically about twice as large as those based on *F*, and *R*- factors based on ALL data will be even larger.

### Fractional atomic coordinates and isotropic or equivalent isotropic displacement parameters (Å^2^)

**Table:**

	*x*	*y*	*z*	*U*_iso_*/*U*_eq_	
Cl1	0.58789 (14)	1.0709 (3)	1.25398 (13)	0.0754 (6)	
O1	0.7358 (4)	0.4112 (6)	0.8320 (3)	0.0689 (13)	
O2	1.0029 (3)	0.3682 (6)	0.1329 (3)	0.0573 (11)	
N1	0.7495 (4)	0.7047 (7)	0.7275 (4)	0.0502 (12)	
H1	0.7447	0.8376	0.7257	0.060*	
N2	0.7810 (4)	0.5948 (7)	0.6283 (4)	0.0493 (12)	
C1	0.6292 (5)	0.9354 (10)	1.1301 (5)	0.0505 (15)	
C2	0.6174 (5)	1.0320 (9)	1.0215 (5)	0.0571 (16)	
H2	0.5886	1.1673	1.0156	0.069*	
C3	0.6487 (5)	0.9259 (9)	0.9215 (5)	0.0559 (15)	
H3A	0.6402	0.9908	0.8484	0.067*	
C4	0.6922 (4)	0.7256 (8)	0.9284 (5)	0.0395 (13)	
C5	0.7063 (5)	0.6364 (9)	1.0389 (5)	0.0561 (16)	
H5	0.7393	0.5039	1.0456	0.067*	
C6	0.6732 (5)	0.7365 (10)	1.1399 (5)	0.0592 (17)	
H6	0.6805	0.6708	1.2128	0.071*	
C7	0.7264 (5)	0.5997 (10)	0.8265 (5)	0.0500 (14)	
C8	0.8292 (5)	0.7001 (9)	0.5487 (5)	0.0482 (15)	
H8	0.8390	0.8418	0.5605	0.058*	
C9	0.8700 (4)	0.6108 (9)	0.4395 (4)	0.0424 (13)	
C10	0.9473 (5)	0.7190 (8)	0.3688 (5)	0.0482 (14)	
H10	0.9700	0.8532	0.3901	0.058*	
C11	0.9914 (5)	0.6360 (9)	0.2693 (5)	0.0504 (15)	
H11	1.0449	0.7116	0.2248	0.060*	
C12	0.9561 (5)	0.4384 (9)	0.2346 (5)	0.0441 (14)	
C13	0.8792 (5)	0.3255 (8)	0.3021 (4)	0.0448 (14)	
H13	0.8558	0.1925	0.2791	0.054*	
C14	0.8365 (5)	0.4103 (9)	0.4045 (5)	0.0492 (15)	
H14	0.7852	0.3329	0.4503	0.059*	
C15	0.9668 (6)	0.1704 (10)	0.0893 (5)	0.076 (2)	
H15A	0.9827	0.0682	0.1496	0.115*	
H15B	1.0125	0.1372	0.0218	0.115*	
H15C	0.8807	0.1718	0.0670	0.115*	
O3	0.7025 (4)	0.1217 (6)	0.6473 (4)	0.0689 (12)	
H3	0.7236	0.2346	0.6745	0.103*	
C16	0.5831 (6)	0.1364 (13)	0.5954 (5)	0.092 (2)	
H16A	0.5772	0.2565	0.5456	0.139*	
H16B	0.5254	0.1481	0.6560	0.139*	
H16C	0.5651	0.0147	0.5491	0.139*	

### Atomic displacement parameters (Å^2^)

**Table:**

	*U*^11^	*U*^22^	*U*^33^	*U*^12^	*U*^13^	*U*^23^
Cl1	0.0776 (11)	0.0881 (14)	0.0620 (10)	−0.0048 (11)	0.0168 (8)	−0.0320 (10)
O1	0.111 (4)	0.035 (2)	0.063 (3)	0.008 (3)	0.018 (2)	−0.002 (2)
O2	0.074 (3)	0.051 (3)	0.049 (2)	0.004 (2)	0.024 (2)	−0.004 (2)
N1	0.069 (3)	0.030 (3)	0.052 (3)	0.004 (2)	0.014 (2)	−0.009 (2)
N2	0.064 (3)	0.035 (3)	0.050 (3)	0.003 (3)	0.011 (2)	−0.006 (3)
C1	0.041 (3)	0.065 (4)	0.046 (4)	−0.003 (3)	0.006 (3)	−0.023 (3)
C2	0.065 (4)	0.044 (4)	0.064 (4)	0.000 (3)	0.018 (3)	−0.009 (3)
C3	0.075 (4)	0.038 (3)	0.056 (4)	0.004 (3)	0.017 (3)	0.001 (3)
C4	0.044 (3)	0.034 (3)	0.040 (3)	0.001 (3)	0.004 (3)	−0.008 (3)
C5	0.053 (4)	0.048 (4)	0.068 (4)	0.014 (3)	0.004 (3)	0.003 (3)
C6	0.055 (4)	0.071 (5)	0.052 (4)	0.009 (4)	0.007 (3)	0.001 (4)
C7	0.054 (4)	0.042 (4)	0.054 (4)	0.004 (3)	0.008 (3)	0.001 (3)
C8	0.058 (4)	0.033 (3)	0.054 (4)	0.001 (3)	0.002 (3)	−0.007 (3)
C9	0.047 (3)	0.038 (3)	0.041 (3)	0.002 (3)	−0.001 (3)	−0.004 (3)
C10	0.056 (4)	0.034 (3)	0.054 (4)	−0.008 (3)	0.000 (3)	0.003 (3)
C11	0.059 (4)	0.045 (4)	0.048 (4)	−0.003 (3)	0.010 (3)	0.007 (3)
C12	0.046 (3)	0.045 (4)	0.041 (3)	0.006 (3)	−0.002 (3)	−0.001 (3)
C13	0.057 (4)	0.035 (3)	0.044 (3)	−0.008 (3)	0.010 (3)	−0.008 (3)
C14	0.060 (4)	0.037 (3)	0.051 (4)	−0.007 (3)	0.009 (3)	−0.001 (3)
C15	0.102 (6)	0.058 (5)	0.071 (5)	0.001 (4)	0.017 (4)	−0.022 (4)
O3	0.095 (3)	0.031 (3)	0.080 (3)	0.005 (2)	0.003 (2)	−0.006 (2)
C16	0.102 (6)	0.087 (6)	0.087 (5)	−0.001 (5)	−0.004 (4)	−0.013 (5)

### Geometric parameters (Å, °)

**Table:**

Cl1—C1	1.736 (5)	C8—H8	0.9300
O1—C7	1.224 (7)	C9—C10	1.384 (7)
O2—C12	1.364 (6)	C9—C14	1.398 (8)
O2—C15	1.419 (7)	C10—C11	1.362 (7)
N1—C7	1.349 (6)	C10—H10	0.9300
N1—N2	1.389 (5)	C11—C12	1.385 (7)
N1—H1	0.8600	C11—H11	0.9300
N2—C8	1.268 (6)	C12—C13	1.375 (7)
C1—C6	1.374 (8)	C13—C14	1.389 (6)
C1—C2	1.383 (7)	C13—H13	0.9300
C2—C3	1.384 (7)	C14—H14	0.9300
C2—H2	0.9300	C15—H15A	0.9600
C3—C4	1.379 (7)	C15—H15B	0.9600
C3—H3A	0.9300	C15—H15C	0.9600
C4—C5	1.383 (7)	O3—C16	1.406 (6)
C4—C7	1.477 (7)	O3—H3	0.8200
C5—C6	1.381 (7)	C16—H16A	0.9600
C5—H5	0.9300	C16—H16B	0.9600
C6—H6	0.9300	C16—H16C	0.9600
C8—C9	1.458 (7)		
			
C12—O2—C15	119.0 (4)	C10—C9—C8	121.1 (5)
C7—N1—N2	119.0 (4)	C14—C9—C8	121.4 (5)
C7—N1—H1	120.5	C11—C10—C9	122.4 (5)
N2—N1—H1	120.5	C11—C10—H10	118.8
C8—N2—N1	115.7 (5)	C9—C10—H10	118.8
C6—C1—C2	120.6 (5)	C10—C11—C12	119.6 (5)
C6—C1—Cl1	120.7 (5)	C10—C11—H11	120.2
C2—C1—Cl1	118.7 (5)	C12—C11—H11	120.2
C1—C2—C3	119.6 (6)	O2—C12—C13	124.2 (5)
C1—C2—H2	120.2	O2—C12—C11	115.9 (5)
C3—C2—H2	120.2	C13—C12—C11	119.9 (5)
C4—C3—C2	121.1 (6)	C12—C13—C14	120.0 (5)
C4—C3—H3A	119.4	C12—C13—H13	120.0
C2—C3—H3A	119.4	C14—C13—H13	120.0
C3—C4—C5	117.6 (5)	C13—C14—C9	120.6 (5)
C3—C4—C7	124.9 (5)	C13—C14—H14	119.7
C5—C4—C7	117.5 (5)	C9—C14—H14	119.7
C6—C5—C4	122.5 (5)	O2—C15—H15A	109.5
C6—C5—H5	118.7	O2—C15—H15B	109.5
C4—C5—H5	118.7	H15A—C15—H15B	109.5
C1—C6—C5	118.4 (6)	O2—C15—H15C	109.5
C1—C6—H6	120.8	H15A—C15—H15C	109.5
C5—C6—H6	120.8	H15B—C15—H15C	109.5
O1—C7—N1	121.6 (5)	C16—O3—H3	109.5
O1—C7—C4	122.1 (5)	O3—C16—H16A	109.5
N1—C7—C4	116.3 (5)	O3—C16—H16B	109.5
N2—C8—C9	123.5 (5)	H16A—C16—H16B	109.5
N2—C8—H8	118.3	O3—C16—H16C	109.5
C9—C8—H8	118.3	H16A—C16—H16C	109.5
C10—C9—C14	117.5 (5)	H16B—C16—H16C	109.5
			
C7—N1—N2—C8	−164.0 (5)	C5—C4—C7—N1	−159.7 (5)
C6—C1—C2—C3	0.9 (8)	N1—N2—C8—C9	178.9 (4)
Cl1—C1—C2—C3	−179.4 (4)	N2—C8—C9—C10	−164.4 (5)
C1—C2—C3—C4	−0.4 (9)	N2—C8—C9—C14	13.1 (8)
C2—C3—C4—C5	−1.5 (8)	C14—C9—C10—C11	−0.7 (8)
C2—C3—C4—C7	179.2 (5)	C8—C9—C10—C11	176.9 (5)
C3—C4—C5—C6	3.1 (8)	C9—C10—C11—C12	1.7 (8)
C7—C4—C5—C6	−177.6 (5)	C15—O2—C12—C13	3.0 (8)
C2—C1—C6—C5	0.6 (8)	C15—O2—C12—C11	−177.5 (5)
Cl1—C1—C6—C5	−179.2 (4)	C10—C11—C12—O2	179.0 (5)
C4—C5—C6—C1	−2.7 (9)	C10—C11—C12—C13	−1.5 (8)
N2—N1—C7—O1	2.0 (8)	O2—C12—C13—C14	179.9 (5)
N2—N1—C7—C4	−179.4 (4)	C11—C12—C13—C14	0.4 (8)
C3—C4—C7—O1	−161.9 (5)	C12—C13—C14—C9	0.6 (8)
C5—C4—C7—O1	18.8 (8)	C10—C9—C14—C13	−0.5 (7)
C3—C4—C7—N1	19.5 (8)	C8—C9—C14—C13	−178.1 (5)

### Hydrogen-bond geometry (Å, °)

**Table:**

*D*—H···*A*	*D*—H	H···*A*	*D*···*A*	*D*—H···*A*
O3—H3···N2	0.82	2.47	3.184 (6)	146
O3—H3···O1	0.82	2.12	2.820 (6)	143
N1—H1···O3^i^	0.86	2.08	2.880 (6)	154

Symmetry codes: (i) *x*, *y*+1, *z*.
